# Psychosocial aspects of work and minor psychic disorders in nursing: use of combined models^*^

**DOI:** 10.1590/1518-8345.2769.3068

**Published:** 2018-11-14

**Authors:** Evelin Daiane Gabriel Pinhatti, Renata Perfeito Ribeiro, Marcos Hirata Soares, Júlia Trevisan Martins, Maria Ribeiro Lacerda, Maria José Quina Galdino

**Affiliations:** 1 Universidade Estadual de Londrina, Departamento de Enfermagem, Londrina, PR, Brazil.; 2 Universidade Federal do Paraná, Departamento de Enfermagem, Curitiba, PR, Brazil.; 3 Universidade Estadual de Maringá, Departamento de Enfermagem, Maringá, PR, Brazil.; 4 Universidade Estadual do Norte do Paraná, Departamento de Enfermagem, Bandeirantes, PR, Brazil.

**Keywords:** Occupational Health, Working Environment, Psychological Stress, Mental Disorders, Health Personnel, Nursing Team

## Abstract

**Objective:**

to analyze the combined use of models for the evaluation of work-related psychosocial aspects and their association with the prevalence of Minor Psychics Disorders among nursing workers.

**Method:**

cross-sectional study with a sample of 285 nursing workers. Data collection was performed through the application of a structured sociodemographic and occupational questionnaire and the Demand-Control-Support, Effort-Reward Imbalance and Self-Reporting Questionnaire. Descriptive analysis and a multiple logistic regression were performed.

**Results:**

the prevalence of suspicion of minor psychics disorders was 32.6%. The dimensions of both models were associated with mental health. The full Effort-Reward Imbalance and Demand-Control and Social Support models predict Minor Psychics Disorders to a greater extent than the combined use of partial models.

**Conclusion:**

it was found that the Effort-Reward Imbalance model captured better the magnitude of the Minor Psychics Disorders in this sample of workers compared to the Demand-Control and Social Support model. However, the concomitant use of the theoretical models revealed unique contributions in the evaluation of Minor Psychics Disorders. Considering the complexity of mental illnesses, it is important that different factors be evaluated.

## Introduction

Work has been the focus of attention of scholars because it is considered a relevant factor both in the onset of diseases and in the well-being of individuals^1^. Psychosocial risks stand out among the risks to which workers are exposed, being recognized as world-wide problems affecting all professions^2^. Such factors can be perceived as the interaction between work and worker, environment, and on the satisfaction with the activity performed and with the conditions of the organization. They can also include the capacity of workers, besides their needs, culture and personal situations^3^. Increasing flexibility and precarious of job conditions, labor intensification and interpersonal relationship problems in the work environment favor these factors^2^. They may play an important role in the health and work performance of workers and their seriousness can be identified in terms of physical and mental health consequences^1^ ^,^ ^4^.

In the occupational environment of nursing, the demands are high. These workers deal with complex situations, time pressure, shortage of personnel and material resources, and increasing demand of high performance in order to guarantee the quality of care. Thus, nursing is a profession that characteristically encompasses a physically and emotionally demanding work structure^5^ ^-^ ^6^. In a recent systematic review, it was evidenced that exposure to adverse experiences at work is a risk factor for mental health^4^.

Two theories have been used to evaluate the psychosocial aspects of work: the Demand-Control and Social Support (DCS)^7^ model and the Effort-Reward Imbalance (ERI)^8^ ^)^ model. The DCS model predicts that individuals exposed to high levels of psychological demand, associated with low levels of control at work, are more likely to develop a high stress, which predispose them to deleterious effects on their health^9^. In contrast, the high control involved in decision making and authority and the greater use of skills can mitigate the consequences of the high demands on the health of those workers^7^. The addition of social support at work by supervisors and peers acts as a moderator of stress, reducing the exhaustion of workers^10^.

The ERI model emphasizes social reciprocity and proposes that high levels of work effort must be accompanied by high levels of reward, be they economic benefits, recognition, promotion prospects, or job security. When the individual experiences an imbalance between high effort and low reward at work, this situation is considered stressful, since this imbalance violates expectations about reciprocity and adequate exchange in social life^8^.

According to the ERI model, in the event that efforts arising from external demands or internal motivations are high and rewards low, unfavorable health outcomes are more likely to occur. The model also predicts that the imbalance between effort-reward will be experienced more often by those who have overcommitment at work because they present a greater need for approval from their colleagues^11^.

Despite the similarities between the two models, considering that both assess psychosocial imbalance at work, they have differences. The DCS model refers to the structural characteristics of the psychosocial environment, emphasizing democracy and the division of work. In turn, the ERI model distinguishes personal and macro social characteristics; it considers the individual’s motivational pattern, as well as reciprocity perceptions, incorporated by salary, esteem and safety at work^11^.

Previous studies have compared the DCS and ERI models or used them in combination to predict cardiovascular risk^12^, burnout^13^, musculoskeletal disorders^14^, self-reported health^15^, and mental health^16^ ^-^ ^17^. However, the authors did not identify studies that evaluated Minor Psychics Disorders (MPD) in nursing, using the combination of the two models.

MPD are used to describe depressive and anxious symptoms characterized by non-specific and non-psychotic clinical conditions. These conditions include symptoms such as insomnia, fatigue, irritability, forgetfulness, difficulty concentrating and somatic complaints - headache, stomach pain, and lack of appetite^18^. Research highlights the negative relation of these aspects with personal and occupational factors, such as satisfaction^19^ and reduced ability to work^20^.

Studies report an improvements in the estimated risk for illness by combining the DCS and ERI models^15^ ^-^ ^16^ and other researchers conclude that there is little evidence to support the use of the models in combination^14^.

In this sense, considering the relevance of the nursing workforce, which corresponds to more than 50% of the population of health workers in Brazil^21^, it is believed that the risk assessment for illnesses in these workers is important. Thus, the purpose of this study was to analyze the combined use of the two models for the evaluation of psychosocial aspects at work and their association with the prevalence of Minor Psychics Disorders among nursing workers.

## Method

This is a cross-sectional study conducted at a public university hospital in Londrina, Paraná, Brazil, with an ca. 300-bed capacity. At the time of the study, the population of this institution was composed by 680 nursing workers. The formula for finite populations was used for calculation of the sample size: n=N.p.q.(Zα/2)^2^/(N-1).(E)^2^+p.q.(Zα/2)^2^. The sample size was calculated based on a pilot study performed with 30 employees of this institution, the prevalence of the outcome was 44%, with a 95% confidence interval and a maximum error of 5%, obtaining an (initial) n of 243 workers. The sample was stratified by professional category and an additional of 20% was included in the n (initial), considering the possibility of refusals and partial answers. The losses resulting from incorrect completion of the questionnaire were not replaced.

The inclusion criteria adopted were: nursing workers who worked for at least one year in order to avoid bias due to occupational adaptation^22^. Those who were on leave or removed for any reason during the collection period, those who were re-qualified to another function, besides those who had returned to work for a period of less than 30 days were excluded, a criterion determined by the instrument used in this study (SRQ-20)^23^.

Data were collected between November 2016 and January 2017 through a questionnaire containing sociodemographic, occupational, psychosocial aspects of work and mental health. The questionnaires were delivered to the workers at the work place during working hours by the study’s first author, after clarifying the objectives of the research.

As dependent variable, MPD were measured by the Self-Reporting Questionnaire (SRQ-20). The instrument has 20 dichotomous questions (yes/no) to track non-psychotic disorders, evaluating depressive, somatic and anxiety symptoms. The cut-off point used for suspected MPD was seven or more positive responses^16^. A study that verified the reliability of the instrument in health workers obtained Cronbach’s alpha of 0.82^24^.

The exposure variable related to psychosocial aspects was evaluated using the ERI and DCS theoretical models. The Brazilian version of the Swedish DCS scale consists of 17 questions, five to assess psychological demand, six to assess control, and six investigate social support. For the categorization of the dimension (high/low), the median was adopted as the cut-off point. Then, the partial model was constructed based on the association of dimensions and the work experience was classified as: active work (high demand and high control); passive work (low demand and low control); low wear (low demand and high control) and high wear (high demand and low control). The dimension social support at work (SSW) was considered in the analysis of the complete DCS; the upper quantile (high social support) was used as a reference category. The Cronbach’s alpha coefficient for the DCS dimensions was as follows: demand (0.79), control (0.67) and social support (0.85)^25^.

The ERI scale, measured from the Brazilian version^26^, is composed of 23 questions: six questions evaluate the effort; 11 the reward; and six the overcommitment. The three dimensions were dichotomized (high/low), having the median as cut-off point. In order to analyze the relations between effort and reward (partial model), the score of each dimension was initially calculated and, afterwards, a ratio was constructed using the formula: e/(r*c), where “e” is the score obtained by the questions about effort; “r” is the score obtained by summing the questions about reward; and “c” is a correction factor (0.545454), considering the number of items in the numerator compared to the denominator (6/11). Values ​​bellow or equal to one indicate a favorable condition, that is, low effort and high reward and values ​​higher than one indicate greater effort spent and lower reward received^11^. Overcommitment (OC) was considered in the analysis of the complete ERI model. Cronbach’s alpha coefficient for ERI dimensions for effort was 0.70, for reward was 0.95, and for overcommitment was 0.86^26^.

Data were analyzed and processed using the Statistical Package for Social Sciences (SPSS) version 20.0, after double typing. Sample characterization was made through descriptive statistics, with measures of central tendency and dispersion for quantitative variables, and absolute and relative frequency for categorical variables.

The Kolmogorov-Smirnov test was applied to check the normality of data. Bivariate analyses were performed to verify the association between the independent variables and MPD. Statistical significance was set at p < 0.20 for the multiple logistic regression analysis.

For the multiple binary logistic regression models (enter method), the following analyses were performed: association between the dimensions of the DCS and ERI models with the outcome; association between each partial and complete model (DCS and ERI) with the outcome; and association between the combined DCS and ERI partial models with the outcome. The group not exposed in any model/dimension was considered as reference category. The crude values ​​of these analyses were then presented, as well as those of analyses adjusted by sociodemographic variables (age, sex, marital status and work shift), considering their potential influences in these aspects. Odds ratio (OR) and their respective confidence intervals (CI 95%) were used to estimate associations. The variables that presented p < 0.05 were significantly associated with the outcome.

This study complies with Resolution 466 of December 12, 2012, having previously been approved by the Research Ethics Committee of the institution (CAAE nº 58056916.0.0000.5231).

## Results

The sample of this study was composed by 285 nursing workers. Of these, the majority were female (75.1%); with a mean age of 45 (± 8.2) years; married/common-law married (67.0%) and with higher education (41.1%). Regarding the occupational characteristics, the category of nursing technician predominated (55.1%); as well as activities performed in the daytime period (56.5%); with a workload of up to 40 hours per week (71.6%). The average performance in the institution was 15 years (SD 9.7) and 76.1% reported not having a second job.

Regarding the suspicion of MPD, there was a global prevalence of 32.6% among the sample investigated, resulting in a higher occurrence in cases of high psychological demand, low control, low social support, high effort, high reward and overcommitment to work.

Regarding psychosocial aspects, the workers presented low psychological demand (57.9%), low control (54.0%), low social support (56.5%), low effort (50.9%), low reward (51.9%) and absence of overcommitment to work (58.2%).

In the DCS partial model, there was a predominance of passive work (43.9%), followed by high demand (25.6%), active work (17.2%) and low work demand (13.3%). In the relation ERI, 79.6% presented high effort-reward imbalance.

Table 1 shows the associations between the DCS and ERI dimensions and the MPD. All dimensions were statistically significant and remained statistically significant even after adjusted for confounding variables. Stronger associations were found between psychological demands (OR 3.58, 95% CI 2.04-6.26) in the DCS model and overcommitment (OR 4.67, 95% CI 2.60-8.38) in the ERI model.

**Table 1:** Crude and adjusted odds ratio according to the dimensions of the Demand-Control Model and Effort-Reward Imbalance Model and Minor Psychics Disorders among nursing workers. Londrina, PR, Brazil, 2016-2017

Model	MPD*		Crude OR^†^ (95%CI)^§^	Adjusted OR^‡^ (95%CI)^§^
	No	Yes		
Demand-control				
Psychological demand				
Low	131	34	1.00	1.00
High	61	59	3.63(2.16-6.11)^||^	3.58 (2.04-6.26)^||^
Control over work				
Low	93	61	2.26(1.34-3.82)^¶^	2.18(1.24-3.83)^¶^
High	99	32	1.00	1.00
Social work support				
Low	96	65	2.32(1.37-3.92)^¶^	2.21(1.25-3.89)^¶^
High	96	28	1.00	1.00
Reward Effort				
Effort				
Low	116	29	1.00	1.00
High	76	64	3.36(1.99-5.69)^||^	3.16(1.79-5.58)^||^
Reward				
Low	117	31	3.12(1.85-5.24)^||^	2.90(1.66-5.04)^||^
High	75	62	1.00	1.00
Overcommitment				
No	135	31	1.00	1.00
Yes	57	62	4.73(2.78-8.05)^||^	4.67(2.60-8.38)^||^

*MPD - Minor Psychics Disorders; †OR - Crude Odds Ratio; ‡OR - Odds Ratio adjusted for age, sex, marital status and work shift; §CI- 95% Confidence Interval; ||p-value < 0.001; ¶ p-value < 0.05

Both partial models were associated with the outcome as presented in Table 2. Workers whose labor conditions were classified as high-demand were 3.60-fold more susceptible to MPD. In addition, those with an inadequate effort-reward relationship were 2.02-fold more likely to have the outcome than those with low imbalance.

**Table 2:** Crude and adjusted Odds Ratio according to the Partial Demand-Control Model and Effort-Reward Imbalance Model and Minor Psychic Disorders among nursing workers. Londrina, PR, Brazil, 2016-2017

Model	MPD*		Crude OR^†^ (95%CI)^§^	Adjusted OR^‡^ (95%CI)^§^
	No	Yes		
DCS partial model^||^				
Low demand	25	13	1.00	1.00
Active work	22	27	0.87(0.49-1.55)	0.89(0.48-1.65)
Passive work	104	21	1.28(0.74-2.21)	1.38(0.76-2.50)
High demand	41	32	3.67(2.17-6.20)^¶^	3.60(2.05-6.34)^¶^
ERI partial model**				
Low imbalance	33	25	1.00	1.00
High imbalance	159	68	1.77(0.98-3.20)	2.02(1.07-3.82)^††^

*MPD - Minor Psychic Disorders; †OR - Crude Odds Ratio; ‡OR - Odds Ratio adjusted for age, sex, marital status and work shift; §CI - 95% Confidence Interval; ||DCS - Demand-Control and Social Support; ¶p-value < 0.001; **ERI - Effort-Reward Imbalance; †† p-value < 0.05

Table 3 presents the multivariate model of the complete models. Stronger associations were found in the complete ERI model (OR 3.76, 95% CI, 1.81-16.41).

**Table 3:** Crude and Adjusted Odds ratio of Minor Psychic Disorders among Nursing Workers, according to the Partial and Complete Demand-Control Model and Effort-Reward Imbalance Model. Londrina, PR, Brazil, 2016-2017

Model	Crude OR* (CI 95%)^‡^	Adjusted OR^†^ (CI 95%)^‡^
Demand-control and social support at work		
DC^§^ and SSW^||^ without exposure	1.00	1.00
Exposure only in DC^§^	1.59(0.63-4.01)	1.67(0.62-4.47)
Exposure only in SSW^||^	2.12(1.13-3.98)^¶^	2.05(1.05-4.00)^¶^
Exposure in DC^§^ and SSW^||^	2.35(1.17-4.70)^¶^	2.15(1.03-4.50)^¶^
Effort-reward and Overcommitment		
ERI** and OC^††^ without exposure	1.00	1.00
Exposure in OC^††^ only	3.88(2.13-7.06)^‡‡^	3.68(1.96-7.29)^‡‡^
Exposure in ERI** only	1.16(0.45-2.99)	1.21(0.44-3.30)
Exposure in OC^††^ and ERI**	2.98(1.75-11.84)^¶^	3.76(1.81-16.41)^¶^

*OR - Crude Odds Ratio; †OR - Odds Ratio adjusted for age, sex, marital status, and work shift; ‡CI - 95% Confidence Interval; §DC - Demand-Control; ||SSW -Social Support at Work; ¶p-value < 0.05; **ERI - Effort-Reward Imbalance; ††OC - Overcommitment; ‡‡p-value < 0.001

In the multivariate analysis of the combined partial models, there was an increase in the strength of association when compared to the reference category, that is, absence of exposure in both models, as shown in Table 4.

**Table 4:** Crude and Adjusted Odds Ratio of Minor Psychic Disorders among Nursing Workers according to the Partial and Combined Demand-Control Model and the Effort-Reward Imbalance Model. Londrina, PR, Brazil, 2016-2017

Model	Crude OR* (CI 95%)^‡^	Adjusted OR^†^ (CI 95%)^‡^
Combined models		
ERI^§^ and DC^||^ without exposure	1.00	1.00
Exposure in DC only^||^	1.53(0.51-4.56)	1.18(0.39-3.53)
Exposure in ERI^§^ only	1.23(0.45-3.35)	1.26(0.38-4.14)
Exposure in ERI^§^ and DC^||^	1.69(0.48-5.91)	1.99(0.51-7.73)

*OR - Crude Odds Ratio; †OR - Odds Ratio adjusted for age, sex, marital status, and work shift; ‡CI - 95% Confidence Interval; **ERI - Effort-Reward Imbalance; §DC - Demand-Control

## Discussion

In this study, most sociodemographic and occupational characteristics resemble other investigations performed with nursing workers. These data reflect a sample of individuals with a predominance of women, with only one job, and a considerably long time of work experience^14^.

The prevalence of MPD found in the present investigation (32.6%) is similar to other studies performed with hospital nursing workers in Bahia (35.0%) and Rio Grande do Sul (33.7%)^20^ ^,^ ^27^. However, these are higher than those found in health workers in general, in which prevalence was identified to be between 17.1% and 21.0%^16^ ^,^ ^19^.

The higher prevalence found in nursing workers may be associated with unsatisfactory working conditions, such as high patient demand, constant contact with suffering and pain, conflicting interpersonal relationships, insufficient human and material resources, intense work rhythms, need for constant improvement in terms of technological and scientific advances, low recognition and valorization, work in shifts, and double or triple work journeys^5^ ^,^ ^28^ ^-^ ^29^.

A study that verified the association between MPD and reduced capacity for work in nursing in Rio Grande do Sul identified that these workers are 2.7-fold more likely to have reduced capacity for work^20^. This finding demonstrates that mental problems not only cause damage to the health of workers but can also result in a worse quality of care provided.

The presence of MPD is in line with studies that indicate that these problems are related to aspects of the work process that include high psychological demand, low control, low social support, imbalance between effort and reward and overcommitment, considered factors associated with stress of workers and mental suffering^16^ ^,^ ^20^ ^,^ ^27^.

Among the assessed dimensions of the DCS model, psychological demand was more closely associated with mental illness. This result is in line with evidence from meta-analyses of longitudinal approach that demonstrate that the risk of a worker developing MPD can be predicted by high psychological demands, followed by low social support and low labor control^30^. Another study carried out with nurses in Scotland found that the demand predicted an increase in the cardiac response at each working day, being considered a good predictor of stress^31^.

The control group in this study had less explanatory power for MPD and this fact may be related to the characteristics of the present sample, composed by more experienced workers. Scholars report that as age and experience, control increases, because these workers feel more confident and this perception of control may have less relevance in their illness^32^.

In relation to the ERI model, in the present study, the overcommitment presented greater magnitude and statistical significance when compared to the other dimensions. This result is corroborated by researches that also found a greater strength of association between these dimensions^15^ ^-^ ^16^. Workers with these characteristics tend to underestimate the demands, taking on excessive workloads and overvaluing their coping abilities, so that they may be more exposed to unfavorable situations, increasing exhaustion^13^ ^,^ ^29^.

Regarding the partial models, in this study, workers classified in the high-demand group and who showed a high effort-reward imbalance presented a greater predisposition to illness, data that are similar to other investigations carried out in Brazil and abroad^13^ ^,^ ^16^ ^,^ ^30^. This result reaffirms what theoretical models postulate, because situations in which individuals experience high psychological demand and ineffective control or high effort in the work and low benefits in terms of salary, possibilities of promotion and positive feedback seem to favor emotional distress, affecting the workers’ physical and mental health^11^ ^,^ ^25^.

In this study, the combination of the partial models, despite increasing the magnitude of association, caused a loss of statistical significance. Moreover, the performance of the complete models was not achieved. Regarding performance, similar data have been identified in national and international studies that have shown that the combination of the DC and ERI models is useful for the evaluation of different stressors. However, this does not overcome the usefulness of the isolated complete ERI model that has shown to be effective in the analysis of health outcomes^13^ ^-^ ^16^ ^,^ ^33^.

One possible explanation for this finding is that the dimensions of social support and overcommitment may capture important issues regarding the health work context, contributing to explain why the performance of the combined partial models did not outweigh the use of the complete models.

In this sense, it is important to emphasize that social support is an essential factor in health work. Such support develops in an environment permeated by interpersonal relationships, especially in nursing, where workers can experience a poor work environment with challenging demands.

As limitations of this research, the little standardization in the evaluation of the exposition of the theoretical models adopted must be mentioned. In the DCS model there are several ways of evaluating exposure variables; in fact, studies have already reported this limiting aspect^15^ ^,^ ^34^. In the ERI model, a similar issue can be pointed out, in which exposure can be categorized by means of a cut-off point greater than one, or by categorizing the ratio in tertiles^11^.

However, this study revealed unique contributions of the models adopted for the evaluation of MPD. In view of the complexity of mental illnesses, it is important to evaluate different factors, among them, high work demands, low control, high effort-reward imbalance, low social support and overcommitment at work.

## Conclusion

The findings of this study showed consistent associations between work demands, levels of control, social support, extrinsic effort, reward, overcommitment, and MPD. The prevalence of suspected MPD was 32.6%. Both the DCS and the ERI model presented strong predictive power.

The results showed that the complete ERI and DCS models predict MPD to a greater degree than the combined use of partial models. Such evidence may be related to the magnitude of association found in the dimensions of social support and overcommitment that are not included in the partial models. The complete ERI model showed a stronger association to MPD compared to the DCS model.

Finally, it is suggested that health institutions invest in support networks, seeking improvement in interpersonal relationships in the occupational environment, as well as enabling strategies that promote professional and personal development, in order to minimize the effects that may interfere in mental illness.
